# Long-term outcome of surgically treated and conservatively managed Rathke cleft cysts

**DOI:** 10.1007/s00701-024-06052-8

**Published:** 2024-04-01

**Authors:** Michael Schmutzer-Sondergeld, Jonathan Weller, Jun Thorsteinsdottir, Christian Schichor, Walter Rachinger, Niklas Thon, Moritz Ueberschaer

**Affiliations:** 1 Department of Neurosurgery, LMU University Hospital, LMU Munich, Marchioninistr 15, 81377 Munich, Germany; 2 Department of Neurosurgery, University Hospital Salzburg, Paracelsus Medical University, Salzburg, Austria

**Keywords:** Rathke cleft cysts, Transsphenoidal approach, Functional outcome, Endocrinological outcome, Secondary cyst progression

## Abstract

**Objective:**

Rathke cleft cysts (RCC) are benign lesions of the sellar region that require surgical treatment in case of visual deterioration or progression of the cyst. However, the natural course is often stable and asymptomatic.

We aimed to investigate the characteristics of patients with cyst progression during follow-up (FU) and to compare the natural history of patients with RCC with patients who underwent surgery.

**Methods:**

Patients with an MR morphologic cystic sellar lesion classified as RCC between 04/2001 and 11/2020 were included. Functional outcomes, including ophthalmologic, endocrinologic, and MRI data, were retrospectively analyzed and compared between surgically treated patients, patients on a “watch and wait” strategy (WWS), and patients on a WWS who underwent secondary surgery due to cyst progression.

**Results:**

One hundred forty patients (median age 42.8 years) with RCC on MRI were identified. 52/140 (37.1%) underwent primary surgery. Of 88 patients (62.9%) with initial WWS, 21 (23.9%) underwent surgery for secondary cyst progression. Patients on the WWS had significantly smaller cyst volumes (*p* = 0.0001) and fewer visual disturbances (*p* = 0.0004), but a similar rate of hormone deficiencies (*p* = 0.99) compared with surgically treated patients preoperatively. Postoperatively patients suffered significantly more often from hormone deficiencies than WWS patients (*p* = 0.001).

Patients who switched to the surgical group were significantly more likely to have preoperative T1 hyperintense signals on MRI (*p* = 0.0001) and visual disturbances (*p* = 0.001) than patients with continuous WWS. Postoperatively, these patients suffered more frequently from new hormonal deficiencies (*p* = 0.001). Endocrine and ophthalmologic outcomes in patients with primary and secondary surgery were comparable.

Multivariate analysis showed that WWS patients were at a higher risk of requiring surgery for cyst progression when perimetric deficits (*p* = 0.006), hyperprolactinemia (*p* = 0.003), and corticotropic deficits (*p* = 0.005) were present.

**Conclusion:**

Surgical treatment of RCC may cause new hormonal deficiencies, which are rare in the natural course. Therefore, the indication for surgery should be carefully evaluated. Hyperprolactinemia and corticotropic deficits were significant indicators for a secondary cyst progression in patients with RCC. However, a significant amount of almost 25% of initially conservatively managed cysts showed deterioration, necessary for surgical intervention.

**Supplementary Information:**

The online version contains supplementary material available at 10.1007/s00701-024-06052-8.

## Introduction

Rathke cleft cysts (RCC) are benign cystic lesions of the sellar region originating from remnants of the craniopharyngeal duct. The incidence is quite high at 22% according to cadaveric studies [23]. However, the rate of symptomatic cysts requiring surgery is relatively low. Only 2–9% of all surgically treated sellar lesions are RCC [19, 28].

Surgery is indicated in space-occupying cysts that lead to worsening visual acuity and visual field loss due to compression of the optic apparatus and may lead to a significant improvement of these symptoms in up to 80% [3, 11, 21]. Surgical complications such as a new hormone deficiency of the anterior or posterior pituitary or leakage of cerebrospinal fluid may occur in about 5–43% of cases [1, 5, 17].

Therefore, incidental asymptomatic findings are often followed by regular MRI controls.

The natural course has previously been described as benign. Nevertheless, cyst progression is reported in about 10%, and/or new hormone deficits can occur over the course [12].

While risk factors for recurrence/progression in surgically treated patients such as preoperative cyst size or squamous metaplasia in the cyst wall and new-onset diabetes insipidus postoperatively have been identified [13, 21, 24], the literature on risk factors for progression in nonsurgical RCC is sparse. Only Kinoshita et al. could show that patients older than 57 years are at an increased risk for cyst progression [12].

Identification of these patients with incidental RCC and increased risk of progression is of paramount importance in determining an optimal follow-up strategy.

In addition, the natural history of patients with RCC undergoing a “watch and wait” (WWS) strategy and the outcomes of surgically treated patients need to be understood in order to weigh the risks and benefits of surgery against the natural clinical course.

Therefore, we aimed to retrospectively analyze and compare the clinical and imaging characteristics of three groups of patients with RCC. Firstly, patients with RCC undergoing WWS. Secondly, patients with progressive RCC on a WWS who underwent surgery for this reason. Thirdly, patients with RCC who underwent primary surgery.

## Methods

### Patient population

**Fig. 1:**
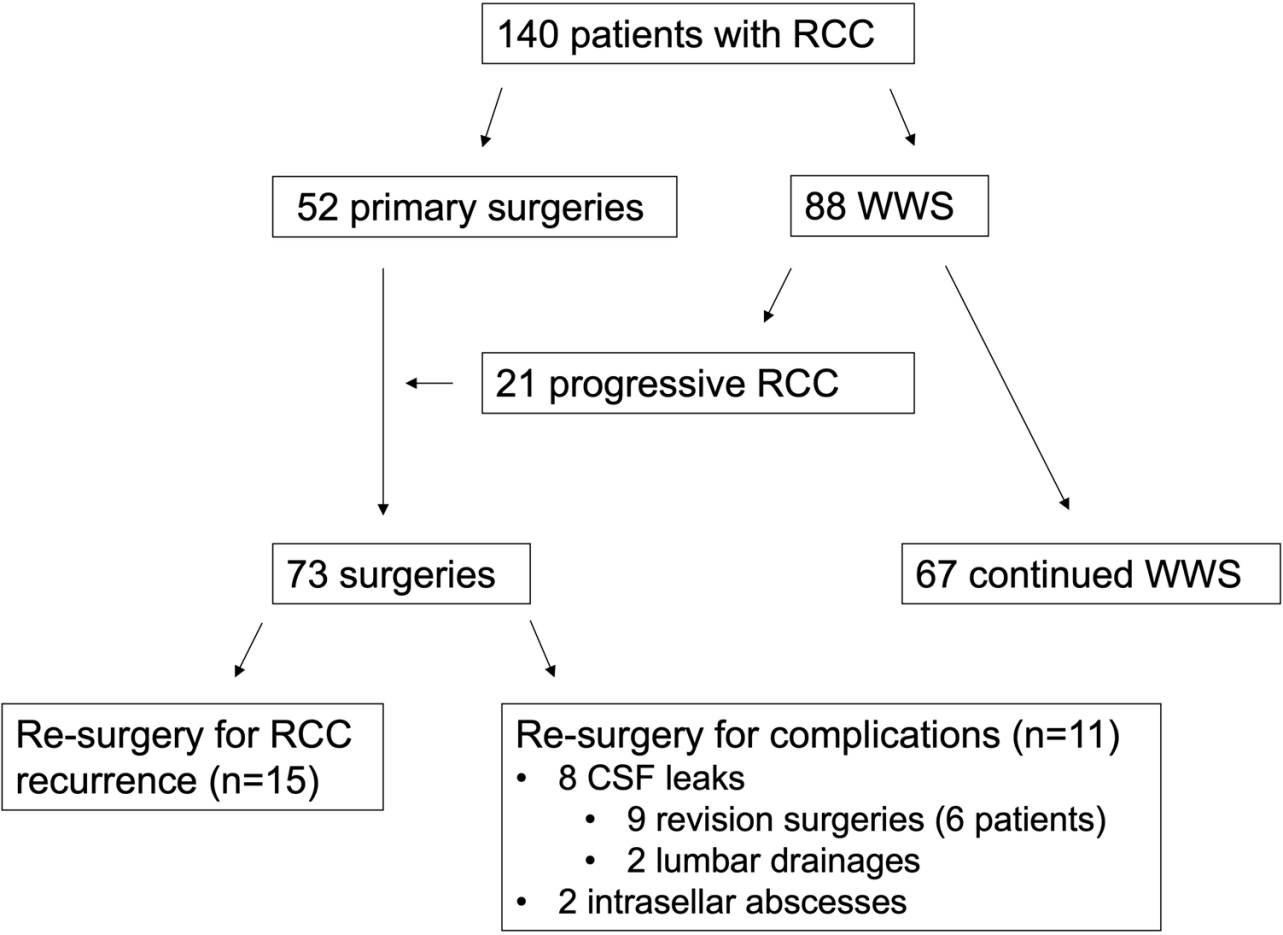
Consort scheme of the patient cohort

The tumor registry of the Department of Neurosurgery of the Ludwig-Maximilians-University in Munich was searched for all patients with MR morphologic diagnosis of RCC who presented between 04/2001 and 11/2020. Initially, two groups were identified, the first group consisting of patients who underwent surgery and the second group consisting of patients who underwent WWS. Then, a third group was established, consisting of patients who underwent surgery after initial WWS (Fig. 1). For surgically treated patients, MR-imaging and clinical data, including laboratory results of pituitary hormone function and ophthalmologic examination results, were assessed preoperatively within 7 days, 6 weeks, 12 months, 24 months, and later after surgery. For functional outcome analysis, outcome parameters were compared with preoperative findings.

For patients undergoing WWS, the same data were collected at the initial admission and at the last FU visit. Data from patients in the WWS group who underwent surgery for cyst progression during the FU period were analyzed in the same manner as data from patients in the surgical group. These patients are referred to as “secondary surgery” patients in the manuscript.

The 73 patients treated surgically for RCC in our study had the diagnosis of RCC confirmed histopathologically. CK7 and 20 were stained in 100% of cases positively.

In the 63 patients in our study who were managed conservatively, diagnosis of RCC was based on cMRI findings.

The study was approved by the local ethics committee of the Ludwig-Maximilians-University in Munich (reference number 21–0271).

### Magnetic resonance imaging (MRI) protocol

Standard MRI (axial T2-weighted sequence with slice thickness of 2 mm, 3-dimensional T1-weighted and constructive interference in steady-state (CISS) sequences with slice thickness of 1 mm) was performed before and after intravenous administration of gadopentetate dimeglumine (Magnevist; Schering Corporation, Kenilworth, NJ) (0.1 mmol/kg body weight) on 1.5- or 3.0-T scanners (Magnetom Symphony, Siemens, Erlangen; Signa HDxt; GE Healthcare, Little Chalfont, UK) with axial, sagittal, and coronal reconstructions. Imaging findings were interpreted as RCC according to the radiological report and the interpretation of the first author, who is a board-certified neurosurgeon. Only patients that were concordantly interpreted as RCC were included. According to previous publications, mainly cystic intra- or suprasellar lesions with or without contrast-enhancing cyst wall with or without the presence of intracystic nodules and/or hemorrhage were considered RCC [4, 26].

Cyst size was calculated by volumetry. Semi-manual segmentation of pre- and postoperative T2/CISS images was performed using the SmartBrush tool of the Elements Brainlab software (Brainlab, Munich, Germany).

An increase in postoperative cyst volume or the initial volume in the conservative group of more than 25% or symptomatic progression of the residual cyst was defined as progression. If the radiologic and neurosurgical evaluation of the postoperative or follow-up MRI images showed a contradictory statement regarding a possible RCC progression or recurrence, a new follow-up MRI image was obtained after 3 months in asymptomatic patients. If postoperative or follow-up imaging did not indicate a residual cyst, any new sellar cystic pathology during FU was considered a cyst recurrence.

### Ophthalmological data

Ophthalmic examinations and tests included visual acuity and visual field perimetry measurements preoperatively/at initial diagnosis (ID) and at specific time points (6 weeks, 12 months, 24 months, and later/last FU) after treatment or during the watch and wait period, as assessed by an ophthalmologist. Deficits in visual acuity were classified as severe deficit (visual acuity 0–0.4), mild deficit (visual acuity 0.5–0.9), and no deficit (visual acuity 1.0), visual field deficits as complete hemianopsia, partial anopsia, or no deficit. A postoperative increase in visual acuity of > 0.2 and/or a decreased visual field defect was defined as an improvement, whereas a postoperative decrease in visual acuity of > 0.2 and/or an increased visual field defect was assumed as deterioration. Otherwise, visual performance was classified as unchanged.

### Endocrinological data and metabolic outcome

In all patients, basal serum levels of growth hormone, insulin-like growth factor 1, adrenocorticotropic hormone, baseline cortisol, prolactin, luteinizing hormone, follicle-stimulating hormone, testosterone and estradiol, thyrotropin, and thyroid hormone (fT3 and fT4) were measured.

In patients undergoing WWS hormone, levels were assessed at first admission and at last FU, in surgically treated patients preoperatively and at 6 weeks, 12 months, 24 months, and later after surgery. In patients who underwent WWS and underwent surgery due to progression of cysts during the course, hormonal status was assessed at initial admission and thereafter in the same manner as in surgically treated patients. Neurohypophyseal function was measured by serum sodium and osmolality levels, fluid intake and output as well as urine specific gravity level. Endocrinological deficits were classified as anterior or posterior hypopituitarism, which includes complete and incomplete insufficiencies, accordingly to the impacted lobe. In the case of partial or complete involvement of both lobes, insufficiency was defined as panhypopituitarism. If hormone replacement was necessary due to endogenously reduced hormone production, the corresponding hormone axis was considered insufficient. Postoperative improvement was assumed in case of complete recovery of at least one affected hormonal axis and at least unchanged status of the remaining axes. A new complete/partial insufficiency of each hormonal axis was classified as deterioration. Otherwise, the endocrinological function was classified as unchanged.

### Treatment protocol

Treatment decisions were invariably made by the interdisciplinary tumor board of the Ludwig-Maximilians-University in Munich. Surgery was usually indicated in patients with visual impairment and a corresponding cyst compressing the optic apparatus. In asymptomatic patients, surgery was offered if the cyst compressed the optic apparatus or if the cyst had increased in size by at least 25%. In these patients, the decision was made on a shared basis according to patient preference and comorbidities. This approach for indicating surgery was used in both the primary and secondary surgery groups. Symptomatic cysts were principally approached via a transnasal, transsphenoidal route. In individual cases, a transcranial approach was chosen based on the patient’s anatomical conditions, especially the cyst configuration and the proximity to the carotid arteries and optic nerves. Conservative treatment of RCC was considered, if patients were asymptomatic and/or the cyst showed no compression of the optic chiasm.

### Risk assessment

Perioperative morbidity rates were derived from medical records, and all medical, neurologic, and approach-related adverse events were assessed and classified as either transient or permanent. Functional morbidity was analyzed separately. Depending on the occurrence of morbidity, the disease courses were classified as complicated or unremarkable.

### Statistical methods

The reference point of this study was the date of first admission to the hospital. The last FU date was November 2020. Overall and recurrence respectively progression rates were specified by number of patients/total case number. Results were tested by using a two-way analysis of variance (ANOVA), Student’s *t*-test, and Fisher’s exact test. For risk factor analyses uni- and multivariate tests were conducted. GraphPad PRISM8.0 software was used for statistical analysis (GraphPad, San Diego, CA, USA). Statistical significance was set at *p* < 0.05.

## Results

### Patient characteristics and study population

One hundred forty patients with RCC were identified with 52 patients (37.1%) undergoing primary surgery. Of 88 patients (62.9%) with WWS, 21 patients (23.9%) underwent surgery for cyst progression during FU (Fig. 1). Mean age of patients with primary surgery was 43.9 ± 19.1 years, with secondary surgery 46.6 ± 21.2 years, and with WWS 40.8 ± 17.1 years (*p* = 0.4). Sex was equally distributed in the three groups, with slightly more male patients undergoing surgery (36.5% vs. 28.6% vs. 25.4%; *p* = 0.4). Mean FU for all surgically treated patients was 55.7 ± 50.0 months. Mean FU for conservatively treated patients was 52.2 ± 38.8 months (*p* = 0.53). Surgery was performed via transnasal transsphenoidal approach in 70 cases (95.9%) and via transcranial approach in 3 patients (4.1%). These 3 patients had RCC which, in addition to an intrasellar portion, also had a correspondingly large supra- and parasellar cystic portion, so that a transcranial surgical approach was chosen to achieve better access to the carotid arteries and optic nerves. The other cysts with suprasellar and intrasellar parts were operated via using a transsphenoidal approach. In 65 patients (89.0%), fenestrations with an augmented cyst wall resection were conducted. In the remaining patients, only cyst fenestration was performed as previously described by our group [21]. Demographic data are shown in Table 1.

**Table 1:** Comparison of patient and imaging characteristics of surgically treated RCC and RCC on a WWS

Parameters	Surgery	Watch and wait	*p*-value
Total, *n* (%)	73 (52.1%)	67 (47.9%)	
Sex, *n* (%)			
Female	48 (65.8%)	50 (74.6%)	0.27
Male	25 (34.2%)	17 (25.4%)	
Age (yrs), mean ± SD	44.6 ± 19.6	40.8 ± 17.1	0.21
FU (mo), mean ± SD	55.7 ± 50.0	52.2 ± 38.8	0.79
PFS (mo), mean ± SD	41.5 ± 35.0	52.2 ± 38.8	**0.0025**
Serum Na^+^ (mmol/l), mean ± SD	139.8 ± 3.8	139.5 ± 4.6	0.71
Prolactin (µU/ml), mean ± SD	879.5 ± 559.5	550.5 ± 399.3	0.34
Cyst localization			
Intrasellar	38 (52.1%)	56 (83.6%)	**< 0.0001**
Intra- and suprasellar	35 (47.9%)	11 (16.4%)	**< 0.0001**
Suprasellar	0	0	0.99
Contrast enhancement	71 (97.3%)	67 (100%)	0.49
Hemorrhagic cyst	21 (28.8%)	9 (13.4%)	**0.038**
T1 hyperintensity	19 (26.0%)	0 (0%)	**< 0.0001**
T1 hypointensity	54 (74.0%)	67 (100%)	
T2 hyperintensity	68 (93.2%)	62 (92.5%)	0.99
T2 hypointensity	5 (6.8%)	5 (7.5%)	
RCC volume (cm^3^), mean ± SD			
Preoperatively/ID	8.2 ± 6.5	3.4 ± 3.0	**< 0.0001**
Postoperatively/FU	1.1 ± 0.2	3.2 ± 3.0	**0.0006**

Significant *p*-values are shown in bold

*ID* initial diagnosis, *FU* follow-up

### Comparison of imaging, symptoms, and functional outcome

#### Surgery vs. WWS

Of 73 patients who underwent surgery, 52 patients were surgically treated after the initial diagnosis and 21 patients after the initial WWS. Mean time to surgery was 19.9 ± 11.1 months in those patients undergoing secondary surgery.

Compared to patients with a WWS, surgically treated cysts were significantly larger (*p* < 0.0001), were more likely to show signs of hemorrhage on MRI (*p* = 0.038) as well as suprasellar growth (*p* < 0.0001), and patients were significantly more likely to suffer from visual deterioration (*p* = 0.0004). In both the surgical group and the WWS group, around 27% of patients reported headaches, which improved significantly over time regardless of the group.

Preoperatively, 33/73 (45.2%) patients suffered from visual field deficits, which improved in 22/33 (66.7%) patients after surgery. Accordingly, surgery led to a significant decrease of cyst size (8.2 cm^3^ ± 6.5 vs. 1.1 cm^3^ ± 0.2; *p* < 0.0001). In the WWS group, only 3/67 (4.5%) patients showed visual field deficits that remained stable in two patients and improved in one patient. The size of the cysts on MRI remained relatively unchanged in the WWS group (3.4 cm^3^ ± 3.0 at first diagnosis vs. 3.2 cm^3^ ± 3.0 at last follow-up, *p* = 0.6).

Preoperatively, there was no difference in hormone deficiencies in both groups. Postoperatively, 14/73 (19.4%) patients developed new hypopituitarism, with 9 patients having partial anterior hypopituitarism predominantly of the corticotropic axis, 1 patient having new-onset diabetes insipidus, and 4 patients having new panhypopituitarism. In contrast, only one patient (1.5%) under WWS developed new partial anterior hypopituitarism (*p* = 0.02), while in another patient under WWS, the initial diabetes insipidus resolved. A detailed presentation of the clinical symptoms of the surgery and WWS group as well as in the further subdivision as primary and secondary surgery groups are listed in Tables 2 and 3. A further presentation of the clinical data can be found in the supplementary tables: patient characteristics of primary and secondary surgery groups (supplementary Table 1), clinical symptoms of secondary surgery group and WWS (supplementary Table 2), and functional outcome data (supplementary Table 3 and 4).

**Table 2:** Comparison of symptoms at first admission and after surgery or at last FU between patients undergoing surgery and patients with WWS

	Surgery, total *n* = 73			Watch and wait, total n = 67		
	Pre-OP, *n* (%)	Post-OP, *n* (%)	*p*-value	ID, *n* (%)	Last FU, *n* (%)	*p*-value
Headache	37 (50.7%)	6 (8.2%)	**< 0.0001**	39 (58.2%)	2 (3.0%)	**< 0.0001**
Trigeminal neuralgia	1 (1.4%)	1 (1.4%)	0.99	1 (1.5%)	0	0.99
Diplopia	16 (21.9%)	3 (4.1%)	**0.0024**	21 (31.3%)	2 (3.0%)	**< 0.0001**
Pituitary hormones						
No deficiency	56 (76.7%)	42 (57.5%)	**0.02**	52 (77.6%)	52 (77.6%)	0.99
Ant. hypopituitarism	14 (19.2%)	23 (31.5%)	0.13	13 (19.4%)	14 (20.9%)	0.99
Post. hypopituitarism	2 (2.7%)	3 (4.1%)	0.99	1 (1.5%)	0	0.99
Panhypopituitarism	1 (1.4%)	5 (6.8%)	0.21	1 (1.5%)	1 (1.5%)	0.99
Visual acuity						
Severe deficit (0–0.4)	3 (4.1%)	2 (2.7%)	0.99	0	0	0.99
Mild deficit (0.5–0.9)	18 (24.7%)	12 (16.4%)	0.31	4 (6.0%)	4 (6.0%)	0.99
No deficit (1.0)	52 (71.2%)	59 (80.8%)	0.24	63 (94.0%)	63 (94.0%)	0.99
Visual field						
Complete hemianopsia	18 (25.0%)	4 (5.5%)	**0.002**	0	0	0.99
Partial anopsia	16 (21.9%)	8 (11.0%)	0.11	3 (4.5%)	2 (3.0%)	0.99
No deficit	39 (53.4%)	61 (83.6%)	**0.0002**	64 (95.5%)	65 (97.0%)	0.99

Significant *p*-values are shown in bold

*ID*, initial diagnosis; *FU*, follow-up; *ant.*, anterior; *post.*, posterior

**Table 3:** Comparison of symptoms pre- and postoperatively between patients with primary and secondary surgery

	Pre-OP			Post-OP		
	Secondary surgery, total *n* = 21, *n* (%)	Primary surgery, total *n* = 52, *n* (%)	*p*-value	Secondary surgery, total *n* = 21, *n* (%)	Primary surgery, total *n* = 52, *n* (%)	*p*-value
Headache	8 (38.1%)	29 (55.8%)	0.2	2 (9.5%)	4 (7.7%)	0.99
Trigeminal neuralgia	0	1 (1.9%)	0.99	0	1 (1.9%)	0.99
Diplopia	3 (14.3%)	13 (25.0%)	0.4	1 (4.8%)	2 (3.8%)	0.99
Pituitary hormones						
No deficiency	16 (76.2%)	40 (76.9%)	0.99	12 (57.1%)	30 (57.7%)	0.99
Ant. hypopituitarism	4 (19.0%)	10 (19.2%)	0.99	7 (33.3%)	16 (30.8%)	0.99
Post. hypopituitarism	0	2 (3.8%)	0.99	1 (4.8%)	2 (3.8%)	0.99
Panhypopituitarism	1 (4.8%)	0	0.3	1 (4.8%)	4 (7.7%)	0.99
Visual acuity						
Severe deficit (0–0.4)	1 (4.8%)	2 (3.8%)	0.99	1 (4.8%)	1 (1.9%)	0.5
Mild deficit (0.5–0.9)	5 (23.8%)	13 (25.0%)	0.99	5 (23.8%)	7 (13.5%)	0.3
No deficit (1.0)	15 (71.4%)	37 (71.2%)	0.99	15 (71.4%)	44 (84.6%)	0.5
Visual field						
Complete hemianopsia	3 (14.3%)	15 (28.8%)	0.2	1 (4.8%)	3 (5.8%)	0.99
Partial anopsia	4 (19.0%)	12 (23.1%)	0.99	4 (19.0%)	4 (7.7%)	0.2
No deficit	14 (66.7%)	25 (48.1%)	0.2	16 (76.2%)	45 (86.5%)	0.3

Significant *p*-values are shown in bold

*ant.*, anterior; *post.*, posterior

### Comparison of imaging, symptoms, and functional outcome

#### Secondary surgery vs. WWS

**Fig. 2:**
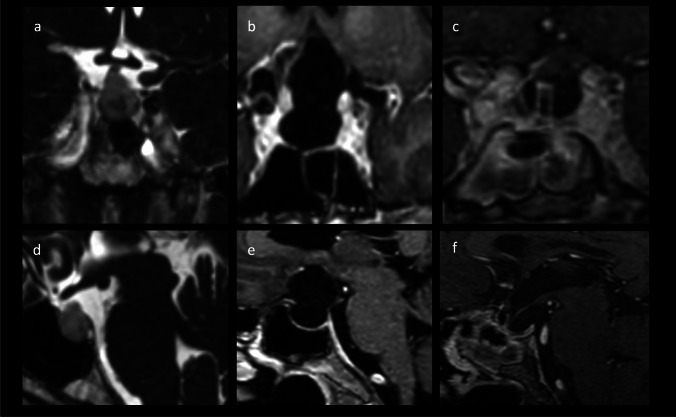
Exemplary case of a patient with cyst progression after initial WWS. **a**, **d** RCC without optic nerve compression at initial diagnosis on MRI CISS sequence. **b**, **e** Contrast-enhanced T1 sequences with evidence of cyst progression 144 months after initial diagnosis. **c**, **f** Postoperative contrast-enhanced T1 sequences without signs of residual cyst

The demographic data of 21 patients who underwent a secondary operation and 67 patients who continued to undergo WWS were comparable. An important marker to determine between the secondary surgery group and WWS was the cyst size, which appeared clearly progressive in the course and extended to the chiasm: cyst size was significantly larger in the secondary surgery group (*p* < 0.0001), and significantly more patients had T1 hyperintense cysts on MRI (*p* = 0.0001). After surgery, the cyst volume decreased to a significantly smaller volume than in patients who continued to undergo WWS (0.8 cm^3^ ± 0.3 vs. 3.2 cm^3^ ± 3.0, *p* = 0.0006). An exemplary case is shown in Fig. 2.

Hormonal status and typical symptoms such as headache and diplopia occurred equally frequently in both groups.

On the other hand, visual field defects were more frequent in patients who underwent secondary surgery than in those who did not (*p* = 0.01). After surgery, visual field deficits and visual impairment improved significantly and were equally common in both groups. However, visual disturbances and partial anopia were still more common even after surgery than in patients who received WWS. While endocrine function was comparable preoperatively, 19.0% suffered from a new partial pituitary insufficiency postoperatively (*p* = 0.0001).

### Comparison of imaging, symptoms, and functional outcome

#### Primary surgery vs. secondary surgery

Demographic data, imaging findings, and preoperative symptoms did not diverge in 51 patients who underwent primary surgery and 21 patients who underwent secondary surgery. The surgical outcome in terms of improvement in vision and hormonal function was also comparable in both groups with 19% of new anterior or posterior hypopituitarism. There was a trend towards better postoperative improvement in pituitary function in patients who underwent primary surgery, but this was not statistically significant (0/21 vs. 8/52 patients; *p* = 0.09).

In a total of 99 surgeries, including 15 surgeries for cyst recurrence and 11 revision surgeries for complications, 8/99 (8.1%) CSF leaks occurred after transsphenoidal microsurgical cyst wall fenestration after a mean period of 53.6 ± 30.7 days. Six out of eight patients underwent in total 9 transsphenoidal microscopical revision surgeries, and 2/8 patients were treated with lumbar CSF drainage. Further, two postoperative intrasellar abscesses (2.0%) after transsphenoidal microsurgery occurred after 205 and 324 days, both of which were surgically revised and subsequently treated with intravenous antibiotics. All these complications occurred in the primary surgery group (transsphenoidal approach) while no perioperative complications were found in the secondary surgery group (*p* = 0.05). All complications are shown in Fig. 1 and listed in Table 4. In 12 (12.1%) cases, transient postoperative electrolyte disorders were diagnosed in the primary surgery group, while only 4 (4.0%) electrolyte disorders were found in the secondary surgery group (*p* = 0.99).

**Table 4:** Complications after primary surgery

Patient number	Sex	Age (years)	Time to revision (days)	Complication	Treatment
1	F	84.2	59	CSF leak	Transsphenoidal revision
2	M	69.8	48	CSF leak	Transsphenoidal revision (2 ×)
3	F	29.1	33	CSF leak	Transsphenoidal revision
4	M	60.7	67	CSF leak	Transsphenoidal revision (2 ×)
5	F	39.0	34	CSF leak	Transsphenoidal revision (2 ×)
6	M	7.1	122	CSF leak	Transsphenoidal revision
7	F	60.6	29	CSF leak	Lumbar drainage
8	M	68.9	37	CSF leak	Lumbar drainage
9	M	51.1	324	Intrasellar abscess	Transsphenoidal revision + i.v. antibiotics
10	F	67.9	205	Intrasellar abscess	Transsphenoidal revision + i.v. antibiotics

*F* female, *M* male, *CSF* cerebrospinal fluid, *i.v.* intravenous

### Indicators for progression during WWS

Univariate analysis revealed that the presence of visual disturbances (*p* = 0.009), and visual field deficits (*p* = 0.0009) was associated with a higher risk of cyst progression (Table 5). Suprasellar cyst localization (*p* = 0.04) as well as corticotropic pituitary deficiency (*p* = 0.02) and hyperprolactinemia (*p* = 0.0009) were also independent indicators for progression. Although T1 hyperintense cysts were significantly more common in patients who underwent secondary surgery, cyst hemorrhage could not be identified as a distinct indicator for progression (*p* = 0.9).

**Table 5:** Indicators for cyst progression in patients on a WWS according to (A) uni- and (B) multivariate analysis

Variable	Odds ratio	95% CI	*p*-value
A) Univariate analysis			
Sex (M vs. F)	1.2	0.3710 to 3.420	0.8
Symptoms preoperatively			
Headache	0.4	0.1559 to 1.189	0.1
Trigeminal neuropathy	0.1	− 0.01107 to 0.04092	0.6
Diplopia	0.3	0.07941 to 1.229	0.1
Visual field deficits	10.7	2.627 to 54.48	0.0009
Decreased visual acuity	6.3	1.606 to 27.42	0.009
Hemorrhagic cyst	1.0	− 1.510 to 1.401	0.9
Cyst localization	3.1	0.03340 to 2.242	0.04
Endocrinological deficiencies			
Corticotropic	7.6	1.376 to 58.60	0.02
Somatotropic	6.9	0.6331 to 154.2	0.1
Thyreotropic	1.4	0.2857 to 5.738	0.6
Gonadotropic	2.6	0.7625 to 8.347	0.1
Diabetes insipidus	3.3	0.1268 to 85.94	0.4
Hyperprolactinemia	6.1	2.141 to 18.38	0.0009
B) Multivariate analysis			
Visual field deficits	2.8	0.1348 to 0.7868	0.006
Decreased visual acuity	0.4	− 0.2919 to 0.4184	0.7
Cyst localization	0.6	− 0.1493 to 0.2777	0.5
Corticotropic deficiency	2.9	0.1404 to 0.7573	0.005
Hyperprolactinemia	3.0	0.09411 to 0.4500	0.003

Significant *p*-values are shown in bold

Visual field deficits (*p* = 0.006) as well as corticotropic pituitary insufficiency (*p* = 0.005) and hyperprolactinemia (*p* = 0.003) were also significant indicators for the progression of the cyst in the multivariate analysis.

## Discussion

This study investigates clinical and radiological data of 140 patients with RCC that were divided into three groups: (1) patients on a WWS, (2) patients undergoing surgery after initial WWS, and (3) patients undergoing primary surgery.

In patients on a WWS, RCC were either asymptomatic or showed stable or even improved symptoms over time, such as headache, diplopia, pituitary dysfunction, and visual disturbances. While most RCC remained stable during FU, approximately 23.9% of patients with WWS experienced cyst progression and subsequent surgical intervention. These patients typically had visual field defects, corticotropic pituitary deficiency, and hyperprolactinemia.

Surgery proved to be effective in improving visual symptoms by reducing the size of the cyst, and outcomes and complications did not differ between patients who underwent primary surgery and those who underwent surgery after initial WWS. The number of new onset partial pituitary insufficiencies of 19% is remarkable, compared to the stable endocrine course of patients with WWS.

Patients’ characteristics such as age and sex distribution with a male to female ratio of 1:2.3 were characteristic of RCC [2, 15]. The mean FU time of 50 months in both the surgical and WWS groups is rather long compared to previous publications on the dynamics of mainly surgically treated RCC, which ranges from 24 to 72 months [1, 2, 6, 7, 12, 17, 20, 22, 25]. According to the standard of care, the microsurgical approach was transnasal, transsphenoidal in 95.9% of cases. As the clinical outcome and recurrence rates after surgery were comparable to previous studies using microsurgical or endoscopic techniques, we do not consider the microsurgical technique a disadvantage of the study [15].

We found a significant difference in cyst volume between the operated and the WWS group. The difference in preoperative RCC volumes reflects that the indication for surgery is usually made for larger cysts. This again confirms the natural course of RCC with the WWS strategy, so that it must be assumed that the smaller volumes generally only rarely lead to complaints. The difference between postoperative RCC volumes and RCC volumes of the WWS group at the last FU demonstrates the efficacy of surgery in reducing RCC volumes.

Headache and endocrine dysfunction were typical symptoms at initial diagnosis, and the frequency was comparable to previous publications [10, 14]. Interestingly, the number of patients with headache was equal in the surgically treated (26.4%) and WWS (27.9%) groups, and both groups experienced significant improvement in headache over time. At the last FU, only 4.3% and 1.4% respectively had persistent headaches. Previous studies have reported improvement in headache with surgery [9, 16] and suggested that inflammatory processes in the cyst wall are responsible for the headache [16]. In view of the significant improvement in headaches in the natural course, we believe that the indication for surgery in patients with headaches must be restrictive. However, targeted studies focusing in particular on the symptom of headache are warranted.

The endocrine course of patients with WWS was very stable throughout the follow-up period, with only 1/67 (1.5%) patients experiencing new anterior pituitary insufficiency. These results are consistent with the findings of Kinoshita et al. [12] describing the natural history of RCC in a large cohort of 229 patients, with 2 patients (0.9%) developing new pituitary hormone deficiency.

In the surgical group, new hormone deficiencies occurred in around 19% of cases, particularly of the corticotropic (*p* < 0.008) and thyrotropic (*p* = 0.057) axis. The rate of new hormone deficiencies after surgery varies widely in the literature between 6% and up to 43% [1, 5, 17, 18, 27]. The aggressiveness of the resection of the cyst wall appears to have a crucial influence on the rate of pituitary dysfunction [1]. Patients in this study were treated with a rather conservative surgical approach with cyst fenestration or augmented cyst wall resection as previously described [21]. The rate of surgical complications was lower in the secondary surgery group, indicating the safety of the surgical procedure after the initial WWS. However, the reason for the lower complication rate remains unclear as there was no difference in demographics, symptoms, cyst configuration, or surgical technique between primary and secondary surgery patients.

The timing of surgery also appears to have no influence on the occurrence of postoperative pituitary insufficiency, as the rates were identical in the primary and secondary surgery groups. An improvement in endocrine function was achieved in 15.5% of patients with primary surgery, which is consistent with previously reported rates [1]. In contrast, no improvement in endocrine function was observed in patients who underwent surgery after initial WWS. The comparison of postoperative pituitary recovery did not differ significantly. However, this aspect should be further investigated in future studies.

Surgery led to a significant improvement of visual field deficits in the primary surgery as well as in the secondary surgery group. Visual acuity also improved after surgery without reaching a statistically significant result (28.8% with mild or severe visual deficit vs. 19.2%; *p* = 0.2). Visual impairment was rare in patients with WWS, with only about 2% of patients having visual field deficits or blurred vision that did not change over time. In these patients, there was no obvious relation between visual disturbances and RCC.

Mixed intensity of RCC on MRI at T1 and T2 was previously described by Eymann et al. [8]. In our cohort, T1 hyperintensity was typical for RCC treated by primary or secondary surgery and was not observed in patients with continued WWS until the end of FU. Accordingly, univariate and multivariate analysis confirmed T1 hyperintensity as a risk factor for secondary surgery after initial WWS.

In addition, hyperprolactinemia or corticotropic pituitary insufficiency might be an early sign of raised intrasellar pressure and lead to secondary cyst progression.

Interestingly, Kinoshita et al. [12] described 6 of 229 patients who developed new symptoms during FU. Apart from two patients with new visual disturbances, there was one patient with new corticotropic insufficiency and one patient with new hyperprolactinemia. This observation seems to be consistent with our results. However, the results need to be verified in a larger cohort of patients in order to enhance the results of the multivariate analysis.

## Conclusion

This study shows the benign natural clinical course of RCC, which is supportive of WWS in RCC without compression of the optic apparatus. However, regular surveillance is warranted as almost 25% of patients with WWS required surgery for cyst progression. T1 hyperintense cysts on MRI, hyperprolactinemia, and hypocortisolism were significant indicators of cyst progression. Delayed surgery after an initial WWS does not lead to a worse outcome than in patients undergoing primary surgery.

## Supplementary Information

Below is the link to the electronic supplementary material.

Supplementary file1 (DOCX 38 KB)

## Data Availability

The original contributions presented in this study are included in the article/supplementary material. Further inquiries can be directed to the corresponding author.
