# Supplementary material for: Long-term outcome of surgically treated and conservatively managed Rathke cleft cysts
Source: Acta Neurochir (Wien). 2024 Apr 1;166(1):159. doi: 10.1007/s00701-024-06052-8 (PMC10984884; doi:10.1007/s00701-024-06052-8)
Supplement: Supplementary file 1 — Supplementary file1 (DOCX 38 KB) [file 701_2024_6052_MOESM1_ESM.docx]

Supplementary Table 1:

Comparison of patient and imaging characteristics of patients who underwent surgery due to cyst progression during WWS (secondary surgery) and patients undergoing primary surgery. Significant p-values are shown in bold.

| *Parameters* | Secondary Surgery | Primary Surgery |  | *p-value* |
| --- | --- | --- | --- | --- |
| Total, n (%) | 21 (28.8%) | 52 (71.2%) | |  |
| Sex, n (%)  Female  Male | 15 (71.4%)  6 (28.6%) | 33 (63.5%)  19 (36.5%) | | 0.6 |
| Age (yrs)  Mean ± SD | 46.6 ± 21.2 | 43.9 ± 19.1 | | 0.6 |
| FU (mo)  Mean ± SD | 40.0 ± 38.3 | 53.7 ± 60.8 | | 0.4 |
| PFS (mo)  Mean ± SD | 38.1 ± 35.0 | 52.2 ± 38.8 | | 0.09 |
| Serum Na^+^ (mmol/l) Mean ± SD | 140.5 ± 1.8 | 137.3 ± 7.5 | | 0.3 |
| Prolactin (µU/ml)  Mean ± SD | 629 ± 595.8 | 982.0± 529.3 | | 0.7 |
| Cyst localization  Intrasellar  Intra- and suprasellar | 13 (52.1%)  8 (47.9%) | 25 (48.1%)  27 (51.9%) |  | 0.3 |
| Contrast enhancement | 21 (100%) | 50 (96.2%) |  | 0.99 |
| Hemorrhagic cyst | 3 (14.3%) | 18 (34.6%) |  | 0.09 |
| T1 hyperintensity  T1 hypointensity | 6 (28.6%)  15 (71.4%) | 13 (25%)  39 (75%) |  | 0.6 |
| T2 hyperintensity  T2 hypointensity | 20 (95.2%)  1 (4.8%) | 46 (88.5%)  6 (11.5%) |  | 0.7 |
| RCC volume (cm^3^)  Mean ± SD  Preoperatively  Postoperatively | 8.6 ± 5.9  0.8 ± 0.3 | 8.6 ± 7.0  1.4 ± 0.6 |  | 0.9  0.4 |

Supplementary Table 2:

Comparison of symptoms at first admission and after surgery or at last FU between patients undergoing secondary surgery and patients with continued WWS (n total=88 of initial WWS). Significant p-values are shown in bold. ID: initial diagnosis, FU: follow-up, ant.: anterior, post.: posterior.

| n total=88 | Secondary surgery  Total n=21 | Continued Watch & wait  Total n=67 | *p-value* | Secondary surgery  Total n=21 | Continued Watch & wait  Total n=67 | *p-value* |
| --- | --- | --- | --- | --- | --- | --- |
|  | **Pre-OP**  **n (%)** | **ID**  **n (%)** |  | **Post-OP**  **n (%)** | **Last FU**  **n (%)** |  |
| Headache | 8 (38.1%) | 39 (58.2%) | 0.1 | 2 (9.5%) | 2 (3.0%) | 0.2 |
| Trigeminal neuralgia | 0 | 1 (1.5%) | 0.99 | 0 | 0 | 0.99 |
| Diplopia | 3 (14.3%) | 21 (31.3%) | 0.2 | 1 (4.8%) | 2 (3.0%) | 0.6 |
| Pituitary hormones  - no deficiency  - ant. hypopituitarism  - post. hypopituitarism  - panhypopituitarism | 16 (76.2%)  4 (19.0%)  0  1 (4.8%) | 52 (77.6%)  13 (19.4%)  1 (1.5%)  1 (1.5%) | 0.99  0.99  0.99  0.4 | 12 (57.1%)  7 (33.3%)  1 (4.8%)  1 (4.8%) | 52 (77.6%)  14 (20.9%)  0  1 (1.5%) | 0.09  0.2  0.2  0.4 |
| Visual acuity  - severe deficit (0-0.4)  - mild deficit (0.5-0.9)  - no defict (1.0) | 1 (4.8%)  5 (23.8%)  15 (71.4%) | 0  4 (6.0%)  63 (94.0%) | 0.2  **0.03**  **0.01** | 1 (4.8%)  5 (23.8%)  15 (71.4%) | 0  4 (6.0%)  63 (94.0%) | 0.2  **0.03**  **0.01** |
| Visual field  - complete hemianopsia  - partial anopsia  - no deficit | 3 (14.3%)  4 (19.0%)  14 (66.7%) | 0  3 (4.5%)  64 (95.5%) | **0.01**  0.05  **0.001** | 1 (4.8%)  4 (19.0%)  16 (76.2%) | 0  2 (3.0%)  65 (97.0%) | 0.2  **0.03**  **0.008** |

Supplementary Table 3:

Comparison of symptomatic outcome between patients with secondary surgery and with WWS. Significant p-values are shown in bold.

|  | Secondary surgery n(%) | Watch & wait  n (%) | *p-value* |
| --- | --- | --- | --- |
| Headache  Improved  Not improved | 6/8 (75.0%)  2/8 (25.0%) | 37/39 (94.9%)  2/39 (5.1%) | 0.1 |
| Trigeminal neuralgia  Improved  Not improved | 0/0  0/0 | 1/1 (100%)  0/1 | 0.99 |
| Diplopia  Improved  Not improved | 2/3 (66.7%)  1/3 (33.3%) | 19/21 (90.5%)  2/21 (9.5%) | 0.3 |
| Endocrine Outcome  Improved  Stable  Worse | 0/21  15/21 (71.4%)  6/21 (28.6%) | 4/67 (5.9%)  63/67 (94.1%)  0/67 | 0.57  **0.01**  **0.0001** |
| Visual Outcome  Improved  Stable  Worse | 4/21 (19.0%)  16/21 (76.2%)  1/21 (4.8%) | 0/67  67/67 (100%)  0/67 | **0.003**  **0.0005**  0.24 |
| Perimetrical Outcome  Improved  Stable  Worse | 5/21 (23.8%)  15/21 (71.4%)  1/21 (4.8%) | 1/67 (1.5%)  66/67 (98.5%)  0/67 | **0.003**  **0.0006**  0.24 |

Supplementary Table 4:

Comparison of symptomatic outcome between patients with secondary surgery and with primary surgery. Significant p-values are shown in bold.

|  | Secondary surgery  n (%) | Primary surgery  n (%) | *p-value* |
| --- | --- | --- | --- |
| Headache  Improved  Not improved | 6/8 (75.0%)  2/8 (25.0%) | 25/29 (86.2%)  4/29 (13.8%) | 0.6 |
| Trigeminal neuralgia  Improved  Not improved | 0/0  0/0 | 1/1 (100%)  0/1 | 0.99 |
| Diplopia  Improved  Not improved | 2/3 (66.7%)  1/3 (33.3%) | 11/13 (84.6%)  2/13 (15.4%) | 0.5 |
| Endocrine Outcome  Improved  Stable  Worse | 0/21  15/21 (71.4%)  6/21 (28.6%) | 8/52 (15.5%)  28/52 (53.8%)  16/52 (30.8%) | 0.09  0.2  0.99 |
| Visual Outcome  Improved  Stable  Worse | 4/21 (19.0%)  16/21 (76.2%)  1/21 (4.8%) | 9/52 (17.3%)  43 (82.7%)  0/52 | 0.99  0.5  0.3 |
| Perimetrical Outcome  Improved  Stable  Worse | 5/21 (23.8%)  15/21 (71.4%)  1/21 (4.8%) | 22/52 (42.3%)  29/52 (55.8%)  1/52 (1.9%) | 0.2  0.3  0.5 |
